# A porcine ligated loop model reveals new insight into the host immune response against *Campylobacter jejuni*

**DOI:** 10.1080/19490976.2020.1814121

**Published:** 2020-09-04

**Authors:** Nicholas M Negretti, Yinyin Ye, Lais M Malavasi, Swechha M Pokharel, Steven Huynh, Susan Noh, Cassidy L Klima, Christopher R Gourley, Claude A Ragle, Santanu Bose, Torey Looft, Craig T Parker, Geremy Clair, Joshua N Adkins, Michael E Konkel

**Affiliations:** a School of Molecular Biosciences, College of Veterinary Medicine, Washington State University, Pullman, WA, USA; b Integrative Omics, Pacific Northwest National Laboratory, Richland, WA, USA; c Department of Veterinary Clinical Sciences, College of Veterinary Medicine, Washington State University, Pullman, WA, USA; d Department of Veterinary Microbiology and Pathology, College of Veterinary Medicine, Washington State University, Pullman, WA, USA; e Produce Safety and Microbiology, United States Department of Agriculture-Agricultural Research Service, Albany, CA, USA; f Animal Disease Research Unit, Agricultural Research Service, United States Department of Agriculture, Pullman, WA, USA; g Washington Animal Disease Diagnostic Laboratory, College of Veterinary Medicine, Washington State University, Pullman, WA, USA; h Food Safety and Enteric Pathogens Research Unit, National Animal Disease Center, Agricultural Research Service, U.S. Department of Agriculture, Ames, IA, USA

**Keywords:** Disease model, innate immunity, proteomics, intestinal disease, pathogenesis

## Abstract

The symptoms of infectious diarrheal disease are mediated by a combination of a pathogen’s virulence factors and the host immune system. *Campylobacter jejuni* is the leading bacterial cause of diarrhea worldwide due to its near-ubiquitous zoonotic association with poultry. One of the outstanding questions is to what extent the bacteria are responsible for the diarrheal symptoms via intestinal cell necrosis versus immune cell initiated tissue damage. To determine the stepwise process of inflammation that leads to diarrhea, we used a piglet ligated intestinal loop model to study the intestinal response to *C. jejuni*. Pigs were chosen due to the anatomical similarity between the porcine and the human intestine. We found that the abundance of neutrophil related proteins increased in the intestinal lumen during *C. jejuni* infection, including proteins related to neutrophil migration (neutrophil elastase and MMP9), actin reorganization (Arp2/3), and antimicrobial proteins (lipocalin-2, myeloperoxidase, S100A8, and S100A9). The appearance of neutrophil proteins also corresponded with increases of the inflammatory cytokines IL-8 and TNF-α. Compared to infection with the *C. jejuni* wild-type strain, infection with the noninvasive *C. jejuni ∆ciaD* mutant resulted in a blunted inflammatory response, with less inflammatory cytokines and neutrophil markers. These findings indicate that intestinal inflammation is driven by *C. jejuni* virulence and that neutrophils are the predominant cell type responding to *C. jejuni* infection. We propose that this model can be used as a platform to study the early immune events during infection with intestinal pathogens.

## Introduction

The initiation of diarrheal disease is a multifactorial process involving the host immune system and the invading pathogen. Understanding the interplay between the host and pathogen is necessary for mitigating the impact of intestinal pathogens on human health. Due to widely varying virulence strategies, some bacterial pathogens cause minimal damage to the cells lining the intestinal tract, whereas other invasive pathogens cause excessive intestinal epithelial cell (IEC) death and severe gut pathology. In either case, an infection may result in an inflammatory response and loss of intestinal barrier function.^1^ The degradation of the intestinal barrier can facilitate the proliferation of the pathogen and potentiate more severe disease symptoms. It is not only the intestinal immune cells that mediate inflammatory responses, but the IECs are also actively involved in intestinal mucosal immunity by producing IL-8 and antimicrobial peptides.^2-4^ Therefore, IECs can be key players in the severity of diarrheal disease.^5^ *Campylobacter jejuni* is a common cause of intestinal illness; however, it is not known what specific host factors respond to the infection, nor is it known how quickly these host responses occur. Our present understanding of disease relies on the appearance of clinical symptoms, which takes several days. In this study, a new methodology has been developed to investigate both bacterial and host responses in an animal model.

*C. jejuni* is a Gram-negative, microaerophilic, curved-rod shaped bacterium that belongs to the epsilon division of the Proteobacteria. This highly motile organism is one of the most common bacterial causes of infectious gastroenteritis in humans. *C. jejuni*-mediated enteritis (campylobacteriosis) is characterized by acute bloody diarrhea, intense abdominal pain/cramps, and fever.^6^ Furthermore, due to molecular mimicry by specific serotypes of sialylated lipooligosaccharide, *C. jejuni* is the most common initiating event of paralyzing autoimmune disorders, including Guillain-Barré syndrome and Miller Fisher syndrome.^7-9^ *C. jejuni* is a zoonotic pathogen because it primarily resides in animals that directly or indirectly transmit the bacterium to humans. *C. jejuni* is particularly effective at colonizing the intestines of chickens, where it can reach bacterial loads in excess of 10^8^ colony forming units (CFU)/gram of cecal contents without causing apparent disease.^6,10^ Consumption of foods contaminated by raw chicken is considered the single most common source of *C. jejuni* infections in humans; however, raw milk, contaminated water, and contaminated produce are also significant sources of infection.^11^

Human infections with *C. jejuni* are often associated with intense inflammatory responses in the intestine. In human infection studies, stools become *C. jejuni* culture-positive 48 hours after bacterial ingestion, and there is, on average, 88.5 hours between ingestion and the onset of diarrhea.^12^ A study that followed a single patient before and after *C. jejuni* infection found that there was a strong induction of IL-1β, IL-8, IL-6, IFN-γ, and C-reactive protein in the serum at 3 to 10 days after onset of symptoms.^13^ *C. jejuni* infection also leads to increased levels of *Campylobacter*-reactive IgA, IgG, and IgM in the blood, and the amount of antibody is correlated with the severity of the infection.^12^ Furthermore, histopathology of intestinal biopsies from infected individuals indicates that there is neutrophil infiltration in the intestine and mucin depletion from the epithelium.^14^ However, it is not known what initiates the inflammatory process. It is possible that either the resident intestinal cells sense the presence of *C. jejuni*, or that the process of cell invasion by *C. jejuni* triggers inflammatory cytokine production. Noteworthy is that *C. jejuni* is not a potent activator of innate immune receptors. For example, the *C. jejuni* flagellum is unique in that it does not activate immune sensing by TLR5. Indeed, the flagellar structure appears to cause the production of the anti-inflammatory IL-10 as a result of its glycosylation.^15^ Furthermore, the *C. jejuni* capsule reduces TLR2 and TLR4 signaling, presumably by masking the lipooligosaccharide.^16^ Due to the invasive nature of *C. jejuni*, we hypothesized that the epithelial cells targeted by *C. jejuni* initiate intestinal inflammation.

The goal of this study was to understand the events that initiate inflammation during *C. jejuni* infection. Specifically, we wanted to dissect the differences in the initiation of inflammation by a virulent *C. jejuni* wild-type strain and a ∆*ciaD* mutant that is significantly reduced in epithelial cell invasion. The *ciaD* gene encodes a secreted effector protein that promotes *C. jejuni* uptake and stimulates IL-8 secretion from cultured epithelial cells.^17^ In previous studies, pigs have proven useful in investigating the pathogenicity of *Campylobacter* due to the anatomical similarity to humans and because pigs develop *C. jejuni*-mediated disease that recapitulates human symptoms. Taylor and Olubunmi^18^ observed inflammatory lesions and shortening of the villous epithelium in the small and large intestines of weaned piglets that resulted from a natural infection with *Campylobacter fetus* subspecies *coli*. Histopathology results from this study were similar to those lesions described in human biopsies from *Campylobacter*-induced enteritis.^14,19^ Babakhani *et al*.^20^ also observed diarrhea with blood and mucus following infection of colostrum-deprived newborn piglets with *C. jejuni*. Histological examination of intestinal biopsies revealed rounding of the mucosal epithelial cells and cell necrosis, as evident from exfoliated cells in the lumen.^20^ These studies illustrate the usefulness of the piglet model in defining the pathogenesis of campylobacteriosis in an immunocompetent animal.

We utilized a pig ligated intestinal loop model to investigate the early events during *C. jejuni* infection and to determine the events leading to intestinal inflammation. This model permits testing of multiple intestinal loops within each animal and provides enough material for analyses of both host and pathogen responses. An additional benefit is that both test and control samples are all within one animal, reducing the confounding effects of animal-to-animal variation. In this study, we investigated the role of *C. jejuni* intestinal cell invasion in host immune stimulation. More specifically, a *C. jejuni* ∆*ciaD* mutant was used because it has identical surface structures as the parental wild-type strain but is lacking a secreted effector protein that potentiates intestinal cell invasion. This study design allowed us to gain a better understanding of why *C. jejuni* infection with an invasive strain and an attenuated mutant results in different clinical outcomes^20^ and to identify the source of the initial immune stimulation. We utilized histopathology, immunoassays, microbiome analysis, and proteomics to evaluate the host response, and we used transcriptomics to analyze the mRNA profile of the *C. jejuni* within the pig intestinal loops.

We found that inoculation of pig intestinal loops with *C. jejuni* results in an inflammatory response as early as 12 hours post-infection, and this was coincident with the appearance of chemotactic and antimicrobial proteins in the intestinal lumen. Based on the identity of the proteins in the intestine at 30 hours after infection, neutrophils were identified as key players during *C. jejuni* pathogenesis. Intestinal permeability was also increased by 30 hours, as evidenced by an increase in the concentration of IL-17A. The appearance of neutrophil-related proteins and histopathological lesions were greater after inoculation with the fully virulent *C. jejuni* wild-type strain than the ∆*ciaD* mutant. This data suggests that the CiaD-mediated process of cellular invasion by *C. jejuni* enhances cytokine production and activated neutrophil influx. Furthermore, detailed proteomic analysis of the intestinal lumen during inoculation with *C. jejuni* revealed numerous antimicrobial proteins that are indicators of intestinal infection and revealed signaling pathways that participate in the development of the disease.

## Results

### Establishment of pig ligated intestinal loops

**Figure 1.**
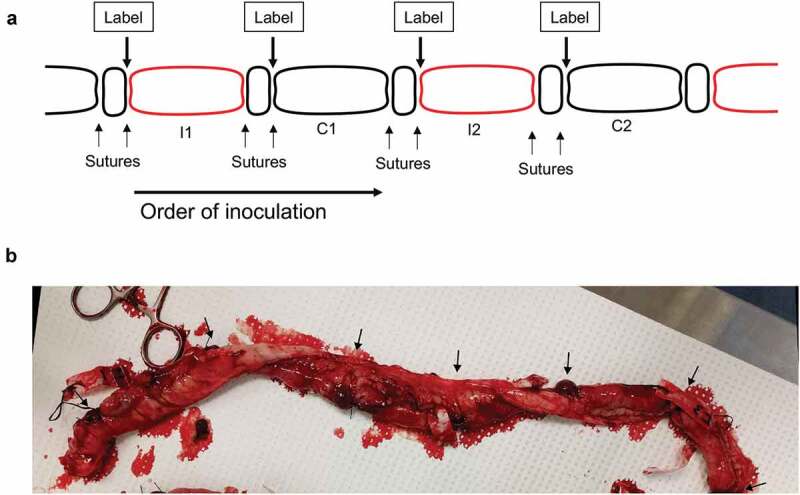
Schematic of the ligated loop creation and representative image of tissue removed from the pig. Panels **a**: Schematic representation of the intestinal pig loops, beginning at the ileocecal junction at the left, and ascending into the small intestine to the right. Loops in red were inoculated with *C. jejuni*, and loops in black are control loops. ‘I’ indicates an infected loop, and ‘C’ indicates a control loop. **b**: Representative tissue removed from the pig at 30 hours post-infection, oriented in the same direction as in Panel A. Arrows indicate the location of the double ligation between each individual intestinal loop.

Minnesota Mini Pigs were obtained from the National Swine Research and Resource Center located at the University of Missouri-Columbia and confirmed to be *Campylobacter* culture negative by plating fecal suspensions on Campy-Cefex agar. Intestinal loops were constructed by double ligating 10-cm sections of the intestine beginning at the ileocecal junction and creating loops toward the jejunum. The ileum, which is the distal portion of the small intestine, was chosen because it has been shown to have the highest microbial load of any location in the small intestine, while still retaining the ability to initiate an innate immune response.^21^ The loops were inoculated in an alternating pattern, where *C. jejuni-*infected loops were separated by a control loop. Two loops in each animal were inoculated with the *C. jejuni* wild-type strain, and two loops were inoculated with a *C. jejuni* ∆*ciaD* mutant.^17^ The intestine was returned to the abdominal cavity, and the pig was maintained under general anesthesia between 3 and 30 hours. Three animals were sampled at 3, 6, 12, and 30 hours after infection. A schematic representation of the ligated loops and a representative image of the intestinal tissue after removal from the animal is shown in Figure 1. Gross pathological differences between infected and control loops were not apparent in the intestinal tissues at any time point. The pigs remained stable over the course of the 30 hours under anesthesia; however, it was necessary to provide a 50% oxygen/50% air mix, as opposed to the more commonly used 100% oxygen, to prevent injury to the lungs. Beginning 4 to 6 hours after anesthesia, it was also necessary to supplement the saline drip with glucose to prevent hypoglycemia. None of the animals developed a fever.

### C. jejuni is active in the intestinal loops

**Figure 2.**
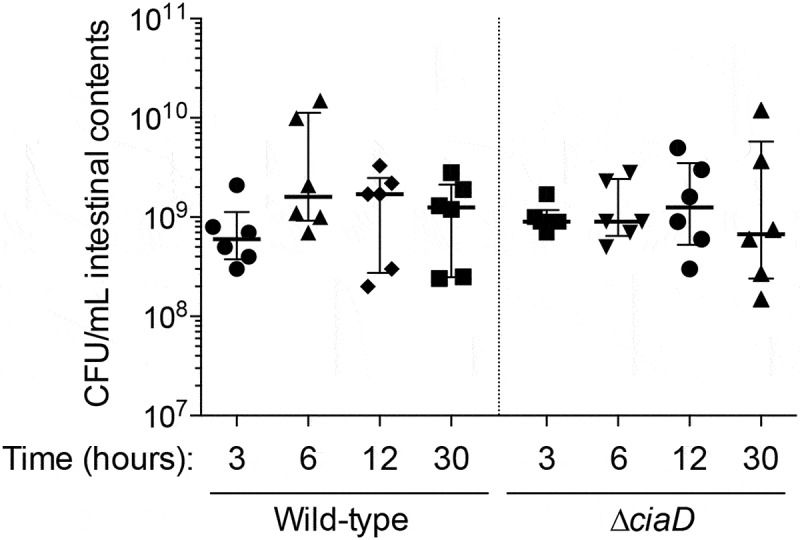
Total colony forming units (CFU) of *C. jejuni* recovered from the pig loops for each of the three experiments. All loops were inoculated with 10 mL of *C. jejuni* between 0.8–4 × 10^9^ CFU/mL in phosphate buffered saline. *C. jejuni* was recovered from the loops at the indicated time points by plating serial dilutions on Campy-Cefex agar. Each dot indicates the CFU/mL from an individual loop. The bars indicate the median ± interquartile range from the indicated samples.

We initially assessed whether the *C. jejuni* in the intestinal loop were metabolically active. Because the intestine rapidly absorbed the liquid used for bacterial inoculation, it was necessary to add 5 mL of PBS to the loops to collect the luminal contents. Intestinal contents were serially diluted and plated on selective Campy-Cefex agar to enumerate the number of *C. jejuni*. Viable *C. jejuni* was not recovered from any of the control loops (limit of detection ~ 10^2^ CFU/mL). For the loops that were inoculated with either the *C. jejuni* wild-type strain or the ∆*ciaD* mutant, the number of *C. jejuni* recovered was always greater than the inoculum, indicating bacterial replication in the intestine. Comparing bacterial abundance over time, the median bacterial load of the *C. jejuni* wild-type strain increased 2.6-fold from 3 to 6 hours, remained stable from 6 to 12 hours, and then decreased ~25% from 12 to 30 hours. The *C. jejuni ∆ciaD* mutant had a similar but delayed trend as compared to the wild-type strain. For the *C. jejuni ∆ciaD* mutant, the median bacterial load in the loops remained unchanged from 3 to 6 hours, increased 1.4-fold from 6 to 12 hours, and then decreased ~50% from 12 to 30 hours (Figure 2). A caveat of these findings is that this is the number *C. jejuni* in the lumen of the intestine and does not account for the tissue-associated bacteria. Overall, these data indicate that the *C. jejuni* were replicating within the intestine. Noteworthy is that the CFU of both the *C. jejuni* wild-type strain and ∆*ciaD* mutant decreased at 30 hours post-infection compared to 12 hours; it is possible that the decrease in recoverable bacteria is due to *C. jejuni* invasion into the tissue, killing by the immune system, or a combination of both.

### The intestinal environment induces transcriptional changes in C. jejuni

Given the indication that the *C. jejuni* were metabolically active and multiplying in the intestinal lumen, we then performed RNA-Seq to determine if virulence gene expression specifically occurred in the intestine. The transcriptome of *C. jejuni* recovered from the intestinal loops was compared with the culture used for inoculation (time zero). While we sequenced samples from every animal, we only acquired sufficient reads (> 10,000) from the 3 and 6 hour samples to determine changes in gene expression. Unfortunately, the 12 and 30 hour samples were not able to be evaluated due to an overabundance of eukaryotic RNA. Nevertheless, to help understand the early signals that *C. jejuni* responds to in the intestinal environment, the data from the pig intestinal loops was compared to *C. jejuni* cultured in MH broth with 0.05% sodium deoxycholate (DOC, previously published data)^22^ and *C. jejuni* cultured with IPEC-J2 cells (immortalized pig intestinal cells). All comparisons were made against the ‘input’ samples grown in MH broth to provide consistency and highlight the contributions of the specific condition. Noteworthy is the *C. jejuni* wild-type strain invades IPEC-J2 cells (not shown). These comparisons allowed us to determine if the responses to the intestinal environment were consistent with either exposure to bile or exposure to cells.

#### Comparing the C. jejuni transcriptome between in vivo and in vitro conditions

**Figure 3.**
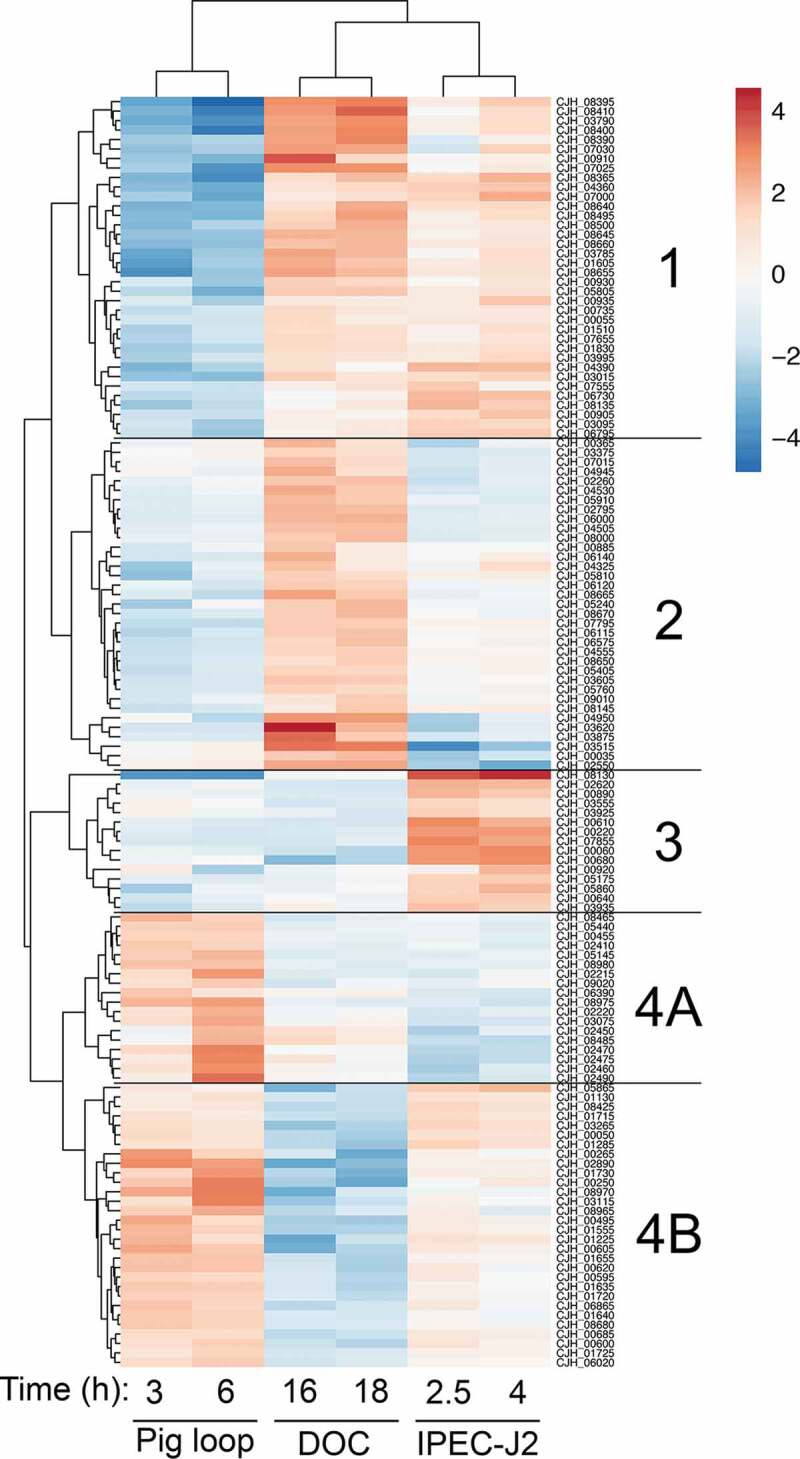
RNA-Seq of the *C. jejuni* in the pig loops indicates similarities and differences to *in vitro* culture conditions. The transcriptome of the *C. jejuni* in the ligated pig loop at 3 and 6 hours post-infection was compared to the transcriptome of *C. jejuni* cultured in sodium deoxycholate (DOC) for 16 and 18 hours and to *C. jejuni* co-cultured with IPEC-J2 cells for 2.5 and 4 hours. In each condition, the samples were compared to the respective ‘input’ sample of *C. jejuni* grown in MH broth. Data was filtered to keep genes that had a Benjamini-Hochberg adjusted *p*-value (*q*-value) of ≤ 0.05, and a log_2_(fold change) of greater than 1.5 (or less than −1.5) in at least four of the six conditions. Hierarchical clustering was applied to the dataset, and this revealed 5 clusters of expression patterns, labeled as 1, 2, 3, 4A, and 4B. In general, the *C. jejuni* cultured with DOC was the least similar to the *C. jejuni* recovered from the ligated pig loop. The *C. jejuni* cultured with the pig intestinal cell line IPEC-J2 had some similarities with both the *C. jejuni* cultured with DOC and recovered from the pig loop. In the diagram, red indicates genes with increased expression and blue indicates genes with decreased expression. The scale bar indicates log_2_(fold change).

Hierarchical clustering was used to evaluate overall trends in the RNA-Seq data (Figure 3) and to help determine what *C. jejuni* was sensing in the intestine. The analysis resulted in the division of the samples into 5 clusters based on similarity of gene expression (labeled 1, 2, 3, 4A, and 4B). A complete list of the genes in each cluster is in Supplementary Table 1. The most apparent trend in the clustering is that pig and DOC-generated samples are markedly different. Most of the genes with increased expression in DOC had decreased expression in the pig, and most of the genes that had lower expression in DOC had increased expression in the pig; the pig and DOC samples have opposite expression patterns. This result may reflect the fact that in the ileum, where the samples were collected, there is a low concentration of bile, as bile is absorbed for reuse along the length of the small intestine.^23^

In contrast to the DOC, *C. jejuni* gene expression within the intestinal loop was similar to the expression during co-incubation with cultured IPEC-J2 intestinal porcine enterocytes. While there were genes regulated in the opposite direction (clusters 1, 3, and 4A, n = 69 genes), there were two clusters of genes that were regulated similarly in the pig and IPEC-J2 cells (clusters 2 and 4B, n = 65 genes). Cluster 2 has decreased expression in both the pig loop and co-incubation with IPEC-J2 cells and is enriched in the COG category “Inorganic ion transport and metabolism” (enriched 5.08 fold, *q*-value = 0.016), which is primarily made up of membrane transporters. Cluster 4B has genes that have increased expression in both the pig loop and IPEC-J2 cells and is enriched in the COG category “Amino acid transport and metabolism” (enriched 3.91 fold, *q*-value = 0.082), which is comprised of the components of the tryptophan biosynthesis pathway. The transcriptome similarity to cultured cells, rather than DOC, indicates that *C. jejuni* likely penetrates the intestinal mucus, and gains access to the epithelial cells by 3 hours post-infection. This finding indicates that the *C. jejuni* encounters signals in the IPEC-J2 cell culture system that are also in the intestine. It is important to note that these signals are not limited to the cells themselves, but also include the components of the tissue culture medium and the relative metabolite and oxygen concentrations.

#### Unique in vivo expression responses induced by the pig

While *in vitro* tissue culture results in similar expression patterns when compared to the intestinal loop, there were genes in clusters 1 and 4A that had unique expression in the *in vivo* environment, which contains elements such as mucus and resident microbiota. Genes in cluster 1 had less expression in the pig compared to IPEC-J2 and DOC, while genes in cluster 4A had higher expression in the pig compared to IPEC-J2 and DOC. The unique genes that were in cluster 1 (lower expression in the pig) were enriched in the COG category “Inorganic ion transport and metabolism” (enriched 10.8 fold, *q*-value = 4.5 × 10^−7^). This category included several iron acquisition genes (*chuA, chuB, chuD, ceuB, cfbpA, cfbpB, cfrA*, and *cj1658*), catalase (*katA*), and the transcriptional regulator *perR*. Cluster 4A (higher expression in the pig) is not significantly enriched in any COG category (*q*-value > 0.6); this category contains several metabolic genes (*sdhA, sdhB, leuA, leuB*), the flagellar component *flgE*, and the adhesin *flpA*. The differential expression of these genes in the pig intestines suggests that the intestinal environment provides cues to the *C. jejuni* that are not present in the *in vitro* conditions.

In summary, we concluded that the *C. jejuni* are not experiencing stress in the initial stages of intestinal colonization at 3 and 6 hours when injected into the intestinal loops. This is based on the fact that iron acquisition systems are not upregulated, in fact, some are downregulated, and there is an absence of reactive oxygen species (ROS) responsive genes. Indeed, a key regulator of ROS stress responses, *perR*, has reduced expression in the pig intestinal loop.^24^ Instead, the *C. jejuni* are increasing expression of genes involved in amino acid synthesis and tissue adherence through the FlpA adhesin during the early stages of intestinal colonization. We hypothesize that the expression of other virulence-related proteins is altered later than 6 hours during infection.

### Initiating the intestinal inflammatory response

#### C. jejuni causes an increase in intestinal IL-8 and TNF-α

**Figure 4.**
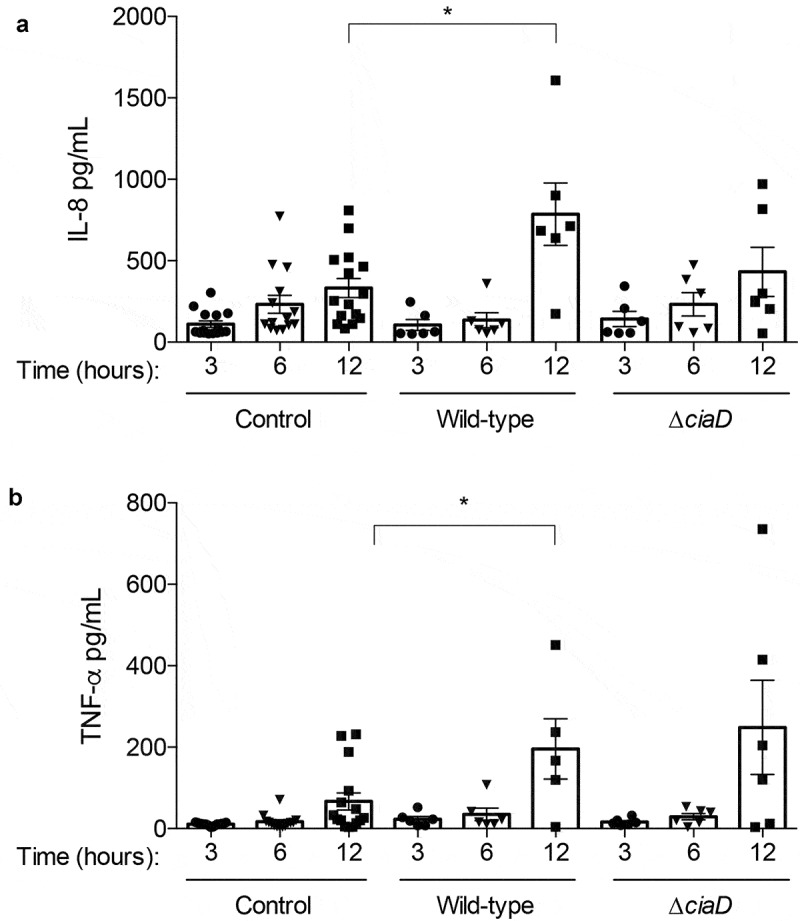
*C. jejuni* causes an increase of intestinal IL-8 and TNF-α. Cytokines in the lumen of the intestinal loops were evaluated by an ELISA. The concentration of each cytokine is presented for each animal, where a single point represents the concentration in a single loop. Panels **a**: The luminal concentration of IL-8 is increased in loops inoculated with the *C. jejuni* wild-type strain at 12 hours post-infection and was not significantly increased by the *C. jejuni* ∆*ciaD* mutant compared to the controls at each time point. **b**: Levels of TNF-α are significantly increased in loops inoculated with the *C. jejuni* wild-type strain at 12 hours post-infection and was not significantly increased by infection with the ∆*ciaD* mutant compared to the controls at each time point. Bars indicate mean ± standard error, * indicates *p* < .05 by one-way ANOVA followed by Sidak’s multiple comparisons test.

A critical question to be answered by this study is whether the initiation of intestinal inflammation was dependent on the ability of *C. jejuni* to invade the IECs. To address this question, luminal contents were evaluated for the presence of acute inflammatory cytokines IL-8 and TNF-α at 3, 6, and 12 hours after infection. Compared to the control loops, we found that the amount of IL-8 in the lumen of the intestine is significantly increased in the loops infected with the *C. jejuni* wild-type strain at 12 hours post-infection. In contrast, the amount of IL-8 in the loops infected with the *C. jejuni* ∆*ciaD* mutant was not significantly increased above the control loops (Figure 4a). The amount of TNF-α in the intestine followed the same trend, where loops infected with the *C. jejuni* wild-type strain had increased amounts of TNF-α at 12 hours post-infection when compared to the controls (Figure 4b). Although not statistically different from the matched control, the average amount of TNF-α at 12 hours post-infection was roughly equivalent between loops inoculated with the *C. jejuni* wild-type strain and the ∆*ciaD* mutant. No correlation was observed with the level of IL-8 or TNF-α and the loop position (i.e., proximal or distal end of the series of loops) (Supplementary Figure 1). Furthermore, no correlation was found between the experimental animal and the level of any of the immune cytokines. These data indicate that the inflammatory cytokine response in the loops is independent of the location of the loop, is reproducible among animals, and is driven by the presence of *C. jejuni*. Based on these results, we concluded that the innate immune response is initiated by 12 hours with the *C. jejuni* wild-type strain, while this induction is attenuated by infection with the ∆*ciaD* mutant. Thus, early intestinal inflammation appears to be related to the ability of *C. jejuni* to invade cells.

#### Kinetic changes in neutrophil protein abundance in the intestine

**Figure 5.**
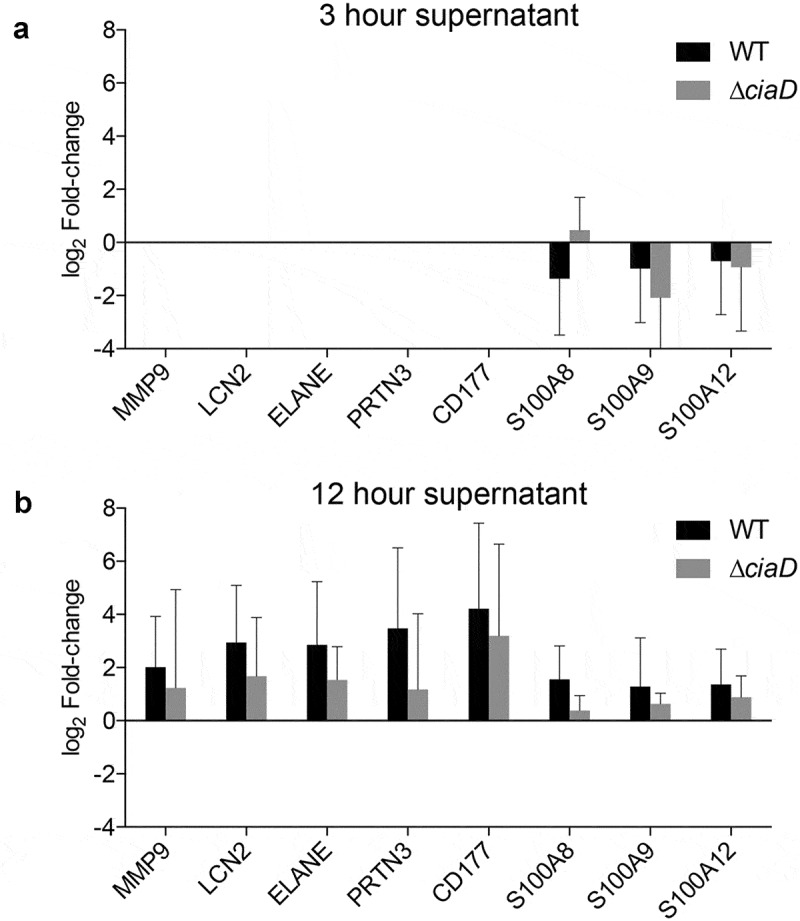
Neutrophil markers increase in the intestinal supernatant in response to *C. jejuni* infection. The proteome from intestinal loop supernatants at 3 and 12 hours was compared to the matched controls at each time point. Initial analysis indicated enrichment of immune related proteins in the intestinal loops, so the specific abundance compared to control loops log_2_ (fold change) of the proteins that make up the neutrophil degranulation pathway were plotted. In general, proteins in the neutrophil degranulation pathway were not detected at 3 hours (Panel a) post-infection but were detected at 12 hours (Panel b) post-infection with *C. jejuni*. Furthermore, the amount of most of the neutrophil markers were higher after a 12 hour incubation period with the *C. jejuni* wild-type strain than with the *C. jejuni* ∆*ciaD* mutant. Error bars indicate standard deviation.

We evaluated the proteome from the supernatant of the intestinal loops to further understand the events occurring in the intestine. The proteome of intestinal supernatants was evaluated at 3 and 12 hours after infection, and the data from infected loops were compared to control loops (Supplementary Table 2). Because the initial analysis suggested that neutrophil markers were abundant in the infected loops, we determined the time frame in which the neutrophil specific proteins appeared. We used CD177 as the primary indicator of neutrophil abundance.^25^ CD177 was not detectable at 3 hours post-infection but was present at 12 hours post-infection in both the loops inoculated with the *C. jejuni* wild-type strain and the ∆*ciaD* mutant. Notably, the amount of CD177 was lower in loops inoculated with the ∆*ciaD* mutant compared to inoculation with the wild-type strain (Figure 5). Additionally, the abundance of CD177 correlated with other neutrophil markers, including matrix metallopeptidase 9 (MMP9), lipocalin 2 (LCN2), elastase (ELANE), and proteinase 3 (PRTN3). These results are consistent with the finding that the *C. jejuni* wild-type strain causes accumulation of both TNF-α and IL-8 in the intestinal lumen 12 hours post-infection, while the ∆*ciaD* mutant induces lower concentrations of IL-8. These data demonstrate that neutrophils are recruited to the site of the infection in a manner that is consistent with the ability of *C. jejuni* to invade cells.

### Early signs of disease in the intestine

#### Histopathological lesions are present in infected loops

**Figure 6.**
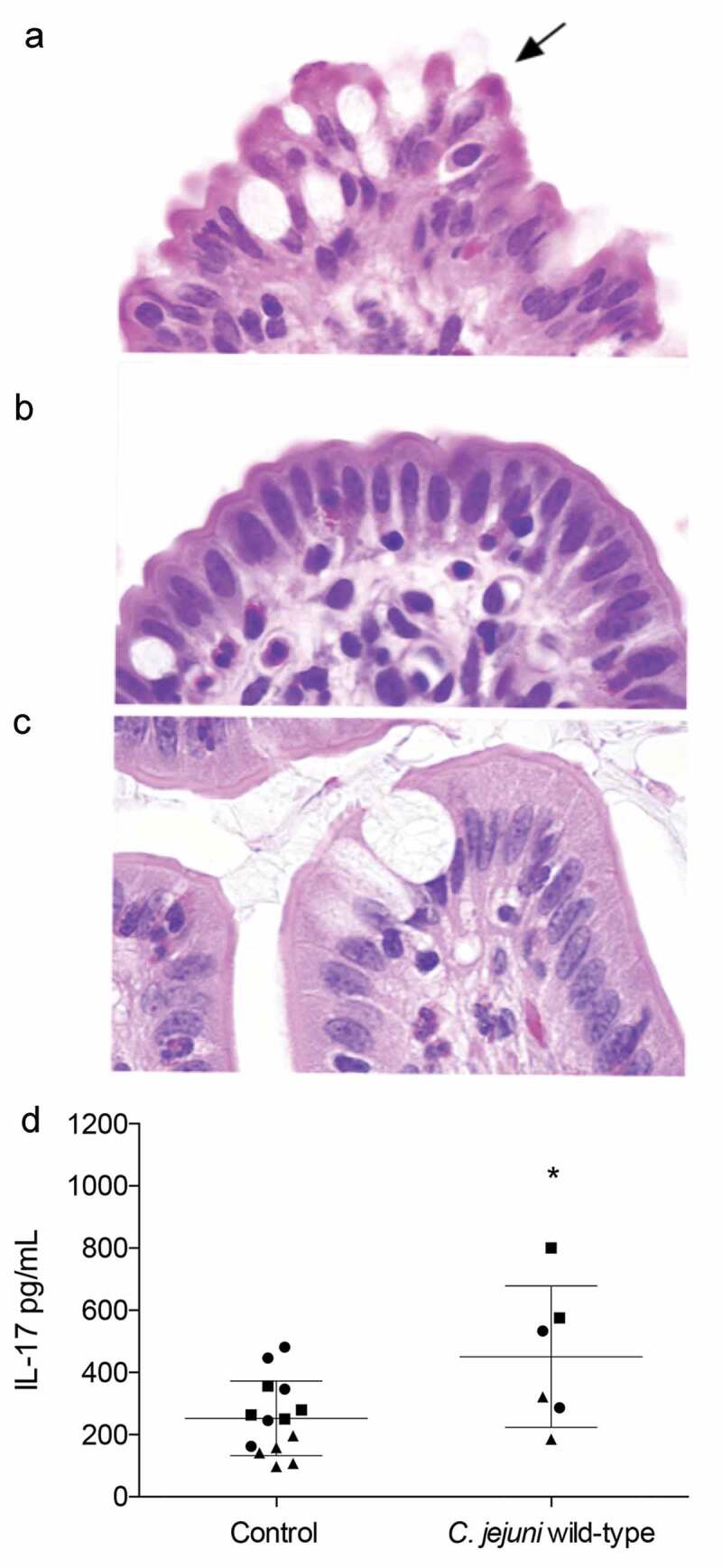
*C. jejuni* infection induces mild lesions and IL-17A production. Intestinal segments at 30 hours post infection were fixed in neutral buffered formalin, sectioned, and stained with hematoxylin and eosin. All images were taken at 100x magnification. Minimal peracute necrosis was observed in the *C. jejuni* infected loops. Panels: **a**: In the loop inoculated with the *C. jejuni* wild-type strain, enterocytes on the villous tips were mildly distorted. Rarely, cells had hypereosinophilic cytoplasm and a pyknotic nucleus (black arrow), indicating necrosis. **b**: In the loop inoculated with the *C. jejuni* ∆*ciaD* mutant, the villous enterocytes were histologically normal. **c**: In the control loop, the villous enterocytes were histologically normal. **d**: Levels of IL-17A were modestly (but statistically significantly) increased in *C. jejuni* wild-type infected loops. Each animal is represented by a different shape, where the circles represent animal 1, squares represent animal 2, and triangles represent animal 3. Bars indicate mean ± standard deviation, * indicates *p* ≤ 0.05 by Student’s *t*-test.

Tissues infected with the *C. jejuni* wild-type strain and non-infected tissues from the three 30-hour experiments were examined for histological changes caused by infection. In the infected loops from two of the three animals, enterocytes at the villous tips were often distorted. Occasional enterocytes were hypereosinophilic and had dense chromatin, indicating necrosis. Associated enterocytes were rounded and dissociated. These markers were not present in the *C. jejuni* ∆*ciaD* mutant-infected nor in the non-infected tissues. Overall, the lesions were consistent with *Campylobacter* infection. These findings support the proposal that damage of the IECs occurs after 30 hours. Representative microscopic sections of the intestinal loops are presented in Figure 6a-c.

#### IL-17A is induced by C. jejuni infection

We tested the intestinal lumen for the presence of IL-17A, which is an immune cytokine indicative of compromised intestinal barrier integrity. At 30 hours after infection, IL-17A was detected at a mean level of 450.59 pg/mL in the loops inoculated with the *C. jejuni* wild-type strain compared to 252.3 pg/mL in the control loops (Figure 6d). The induction of IL-17A in infected loops indicates that there is a specific and localized response to *C. jejuni* infection. Furthermore, this finding also indicates that there is a significant delay between the appearance of *C. jejuni* in the intestine and the beginning of intestinal barrier disruption. This is consistent with the finding that humans become culture positive in the stool approximately 40 hours before the onset of diarrheal symptoms.

#### Intestinal microbiota is consistent among animals

**Figure 7.**
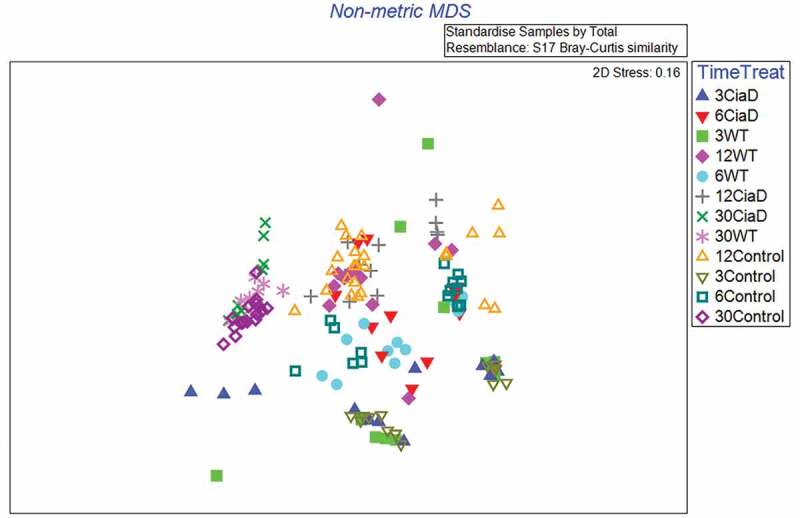
Microbiota in the ligated intestinal loops is insensitive to *C. jejuni* infection. The microbiota of the luminal contents and mucosal scrapings were analyzed by deep sequencing the 16S rRNA gene. Because the addition of *C. jejuni* was a known experimental manipulation in the loops, we removed the *C. jejuni* reads from subsequent analysis so we could observe changes in the resident microbiota. The data below is summarized in a non-metric multidimensional scaling (MDS) plot where data from each individual loop is plotted as a single point. Points are differentiated by both time of incubation (in hours) and infection state. From this data, treatment with *C. jejuni* does not meaningfully change the microbial composition when compared to the control at the same time point, however, the duration of incubation does alter the microbiome. Because each individual animal was assayed on independent days, the data also indicates that the microbiota among the animals was consistent.

Shifts in intestinal microbiota are frequently associated with disease states, so we determined the total microbiome of the intestinal loops from every time point by 16S rRNA gene sequencing. Approximately 50% of the reads from the infected samples originated from *C. jejuni*. Because this was a known experimental manipulation, we compared the microbiome from the *C. jejuni*-infected loops, after the removal of the *C. jejuni* sequence reads, to that of the non-infected loops. The microbiome findings are summarized in Figure 7. No significant differences were observed in the richness or diversity measures of the microbiota between the *C. jejuni-*infected and non-infected control loops at any given time point. Primarily, the observable differences reflected the incubation time and not the presence/absence of *C. jejuni* in the loop. We sampled both the luminal contents and mucosal scrapings for microbiota analysis. While there were small differences in the microbial composition between the mucosal and luminal samples, they largely reflected the same trends in that there were no differences between infected and non-infected loops at a given time point. Importantly, the microbiome data indicated that the microbial communities in the intestine of the three experimental animals at any given time point were consistent. Because these experiments were conducted on different days from individually housed animals, this provides support that the experimental results were not due to varying microbiota among the animals.

### Evaluation of the intestinal proteome

#### Proteomic analysis of the intestinal loops identifies immune markers

We sought to characterize the intestinal proteome in response to the invasive *C. jejuni* wild-type strain and noninvasive ∆*ciaD* mutant early in infection. Samples from the lumen of the intestine were evaluated using a label-free quantitative proteomic approach (Supplementary Table 3). In all of the analyses, the *C. jejuni-*inoculated loops were compared to the control, non-inoculated, intestinal loops at the same time point. Because the intestine is a complex tissue, it was not possible to determine protein abundance changes within a single cell type (i.e., epithelial cells), but the appearance of specific proteins was used to identify specific cell types (i.e., neutrophils) and broad trends in protein abundance.

#### Immune pathways involved in C. jejuni infection

**Figure 8.**
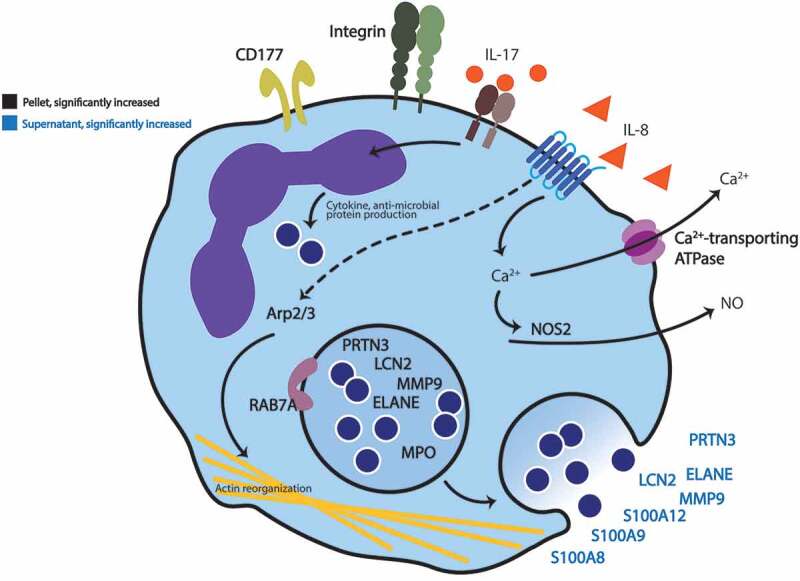
Graphical summary of proteomic analysis. Proteomic analysis of the intestinal loops at 30 hours post infection indicates infiltration of neutrophils. Luminal contents from ligated intestinal loops that were infected with *C. jejuni* were separated into pellets and supernatants by centrifugation, the proteome of each was determined, and then compared to non-infected loops. Proteins indicated on this model were significantly increased in abundance (*p*-value of ≤ 0.05 and a log_2_ fold-change > 0) in intestinal loops infected with *C. jejuni* compared to non-infected loops. The proteins identified are broadly defined as participating in neutrophil function and include: Integrins (α_M_β_2_), CD177, Arp2/3, RAB7A, and NOS2. Additionally, neutrophil-related secreted proteins were detected: S100A8, S100A9, S100A12, LCN2, MMP9, PRTN3, and neutrophil elastase (ELANE). Bold black text indicates proteins found in the pellets collected from the intestine, and bold blue text indicates proteins found in the intestinal supernatant. Non-bold text indicates that the proteins were identified by immunoassay (IL-8 and IL-17A) or are included for clarity and were not directly identified (Ca^2+^ and NO).

The entire proteome from the intestinal lumen after 30 hours of infection was analyzed with the Reactome pathway database^26^ to determine pathway enrichment and guide our analysis. Proteins from both the supernatant and pellet that had a paired *p*-value of < 0.05 and a log_2_(fold change) > 0 were combined and analyzed to discover the changes occurring in response to *C. jejuni*. The analysis was limited to proteins increased in abundance, as we reasoned that these proteins would reveal the host factors that were induced in response to *C. jejuni*, or the cell types recruited to the areas of infection. From this list of proteins, the top enriched Reactome pathway was ‘Neutrophil Degranulation’ (*q*-value = 0.0016), which included the CD177 protein, a surface glycoprotein predominantly present on neutrophils. The amount of the S100A8, S100A9, and S100A12 proteins also increased; these three proteins comprise up to 35% of the cytosolic protein content of a neutrophil and are potent neutrophil chemoattractants.^27^ Further evidence of neutrophil infiltration was the increase in the proteins MMP9, LCN2, ELANE, MPO, and PRTN3 in the lumen of the *C. jejuni-*inoculated loops compared to the uninoculated loops (Figure 8, Supplementary Figure 2). These proteins are associated with neutrophils and have antimicrobial activities. In total, proteomic analysis has allowed for the development of a model revealing that *C. jejuni* triggers the influx of neutrophils to the intestine early in infection, and that this process depends on the *C. jejuni* secreted effector protein CiaD. These findings highlight multiple antimicrobial proteins involved in iron sequestration and bacterial killing in the intestine, including lipocalin-2 and myeloperoxidase.^28,29^ This data also suggests that once neutrophils are present, further neutrophil recruitment could occur via the S100A8 and S100A9 proteins, which are strong chemoattractants.^30^

In addition to immune-related proteins, components of the Arp2/3 complex, which facilitate actin rearrangement, were increased in abundance in the *C. jejuni-*inoculated loops. One of the key requirements for *C. jejuni* invasion of epithelial cells is activating the actin rearrangement and adhesive machinery. While the specific cell-type producing these proteins is not known, it is known that *C. jejuni* relies on actin reorganization to invade epithelial cells.^31^ However, given the apparent abundance of neutrophil-related proteins, an increase in actin remodeling proteins could also be a result of neutrophil migration into the intestine. This influx of neutrophils, and their killing associated antimicrobial proteins, was observed at the 12 hour and 30 hour time points. Thus, we propose that neutrophils drive intestinal inflammation and the initiation of diarrhea in *C. jejuni-*infected individuals.

### Determining the role of epithelial cells in initiating immune responses

#### C. jejuni induces IL-8 in cultured epithelial cells in a CiaD dependent manner

**Figure 9.**
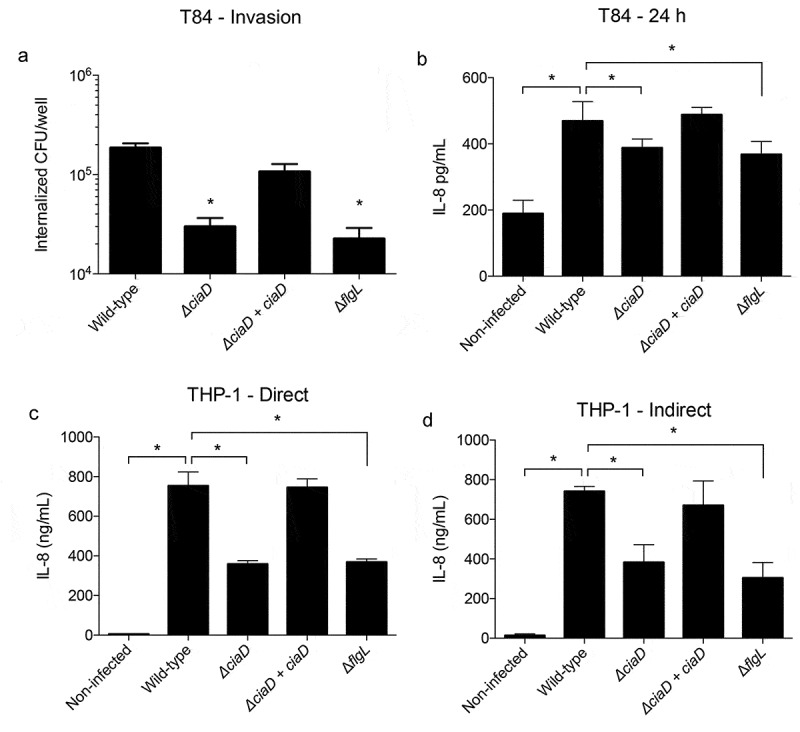
Trends in immune cytokine production are consistent between the pig model and cultured human cells. Panels: **a**: The invasion potential of *C. jejuni* into cultured human colonic (T84) cells was tested using a gentamicin protection assay. T84 cells were infected with a *C. jejuni* wild-type isolate, a ∆*ciaD* mutant, a complemented ∆*ciaD* mutant, and a ∆*flgL* mutant (negative control). The number of bacteria surviving gentamicin treatment (which kills extracellular bacteria) was counted and plotted. **b**: The amount of IL-8 induced by *C. jejuni* from T84 cells was measured after 24 hours of incubation. **c**: Human macrophage-like (THP-1) cells were infected with *C. jejuni* (direct infection) and the amount of IL-8 produced was measured after 24 hours. **d**: Cell-free conditioned medium from infected T84 cells (panel B) was used to treat THP-1 cells, and the amount of IL-8 induced was measured. It was found that the cell-free medium induces similar amounts of IL-8, as direct infection with *C. jejuni*. Bars indicate mean ± standard deviation, * indicates *p* < .05 by one-way ANOVA followed by Sidak’s multiple comparisons test.

The neutrophils detected in response to *C. jejuni* inoculation may have been recruited by chemoattractants produced from resident intestinal cells. To determine a possible source of the initial chemoattractants and to understand if this process happens in human cells, we tested the ability of *C. jejuni* to induce IL-8 from intestinal epithelial cells and macrophage-like cells. To take into account the role of cellular invasion in this process, we first tested if the *C. jejuni* were capable of invading human T84 (immortalized colon cells) cells using a gentamicin-protection assay. We found that the invasion of *C. jejuni* into T84 cells was dependent on the presence of CiaD, as the invasion of the ∆*ciaD* mutant was significantly less than the wild-type strain (Figure 9a, *p* < .05). The amount of invasion by the ∆*ciaD* mutant was equivalent to ∆*flgL* mutant, which lacks motility, a functional flagellar secretion apparatus, and does not invade at all (negative control). Complementation of the ∆*ciaD* mutant restored invasiveness in T84 cells. Furthermore, the amount of IL-8 secreted from the T84 cells was evaluated after a 24 hour incubation period (Figure 9b). The trends in the IL-8 production were similar to the invasion trends, where the *C. jejuni* wild-type strain resulted in more IL-8 in the supernatant than the ∆*ciaD* and ∆*flgL* mutants (*p* < .05). This indicates that *C. jejuni* invasion of the T84 cells participates in IL-8 production, which can promote the recruitment of neutrophils.^32^

#### Secreted factors from epithelial cells can induce IL-8 from THP-1 macrophage-like cells

To test if there were differences in the responses of epithelial cells and immune cells to *C. jejuni*, we infected differentiated THP-1 cells, which are macrophage-like cells that are commonly used to model human monocytes. THP-1 cells were directly infected with the *C. jejuni* wild-type strain, ∆*ciaD* mutant, and ∆*flgL* mutant for 24 hours, and secreted IL-8 was measured (Figure 9c). The trends observed were similar to that of T84 cells, where the wild-type strain resulted in a greater level of IL-8 than both the ∆*ciaD* and ∆*flgL* mutants, and the amount of IL-8 induced by the ∆*ciaD* and ∆*flgL* mutants was roughly equivalent. Complementation of the ∆*ciaD* mutant restored secretion of IL-8 compared to infection with the *C. jejuni* wild-type strain. As would be expected from immune cells, the quantity of IL-8 secreted from THP-1 cells is significantly higher than T84 cells, with secretion in the ng/mL range rather than the pg/mL range. These findings indicate that both epithelial cells and resident immune cells may act together to recruit neutrophils to the intestine.

To determine the extent by which epithelial cells and macrophages work together to promote inflammatory cytokine secretion, cell-free conditioned medium from T84 cells incubated with (or without) *C. jejuni* for 24 hours was added to THP-1 cells. The bacteria-free supernatant (no culturable *C. jejuni*) was able to stimulate the same responses in the THP-1 cells as direct infection with *C. jejuni*, suggesting that the *C. jejuni*-infected human T84 epithelial cells are capable of secreting factors that initiate IL-8 secretion from THP-1 cells (Figure 9d). This finding supports the proposal that *C. jejuni* does not need to directly interact with tissue-resident immune cells to promote IL-8 secretion, and that this process is correlated with the ability of *C. jejuni* to invade the epithelial cells. In total, this data is consistent with the data collected from the pig intestinal loops, where the *C. jejuni ∆ciaD* mutant causes less immune cytokine production and raises the possibility of an epithelial cell-driven mechanism of immune activation.

## Discussion

**Figure 10.**
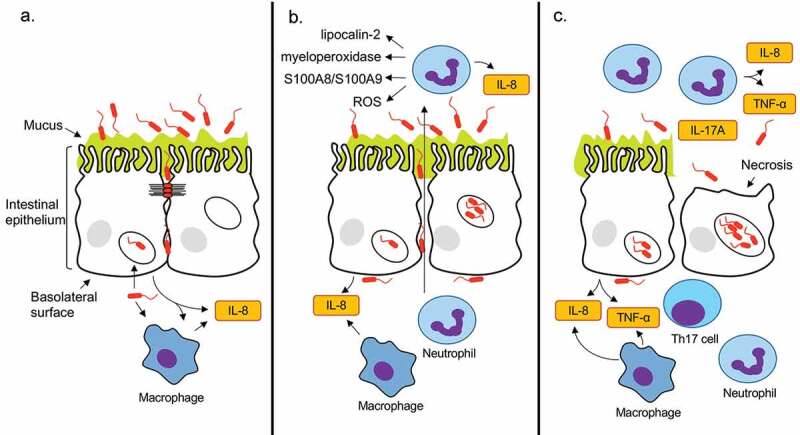
*C. jejuni-*mediated intestinal inflammation is driven by epithelial cell invasion. Panels: **a**: After penetrating the intestinal mucus, *C. jejuni* translocate to the basolateral surface of the epithelial cells by transiently disputing the tight junctions with the secreted protease HtrA. *C. jejuni* then invades the epithelial cells and triggers interleukin 8 (IL-8) secretion at the basolateral surface of the epithelial cells. Resident monocytes (macrophages) are also stimulated both directly and indirectly to secrete IL-8. **b**: Due to the elevated levels of IL-8, there is activated neutrophil influx to the lumen of the intestine. Antimicrobial neutrophil proteins, including lipocalin-2, myeloperoxidase, S100A8, S100A9, and an increase in reactive oxygen species (ROS) kill *C. jejuni* that are not internalized within epithelial cells. As the intestinal inflammation proceeds, intestinal barrier disruption is initiated, permitting more *C. jejuni* access to the basolateral surface where invasion occurs. **c**: As the infection continues, the amount of luminal TNF-α increases and *C. jejuni* multiply within intestinal epithelial cells resulting in necrosis and increased intestinal barrier disruption, as evidenced by the appearance of IL-17A. Ultimately, these processes are potentiated by *C. jejuni* cell invasion and lead to the development clinical symptoms in a host. Cytokines are indicated in yellow boxes.

An animal model for a human disease is fundamental to understanding the disease process and dissecting the pathogen-host interaction when it is not practical to do so in humans. In this study, we have used a pig ligated intestinal loop model to gain insight into the development of *C. jejuni*-mediated disease and to understand the events that initiate inflammation during *C. jejuni* infection. This model was chosen because the symptoms and pathologies that develop in pigs mimic human campylobacteriosis. We have found that *C. jejuni* replicate within the pig intestine, trigger the production of immune cytokines, and cause an influx of neutrophils. This process begins with a motile *C. jejuni*. The flagellum is essential for motility, however, unlike most other bacteria, the *C. jejuni* flagellum does not activate the TLR5 receptor.^15,33^ In combination with chemosensing proteins, there is directional motility of *C. jejuni* toward mucus and epithelial cells.^34-37^ *C. jejuni* reaches the basolateral surface of the epithelial cells by migrating between the cells (paracellular transmigration) utilizing the HtrA protease, which is secreted into the surrounding space and deposited in outer membrane vesicles. The HtrA protease cleaves the cellular tight junction proteins occludin and E-cadherin.^38-40^ *C. jejuni* then uses the CadF and FlpA adhesins^41,42^ to bind fibronectin on the basolateral surface of the cells.^43,44^ *C. jejuni* invade the IECs in a manner that is dependent on secreted effector proteins, including the Cia proteins and FedA.^17,45-48^ In particular, CiaD facilitates cellular invasion by activating the MAPK pathway, leading to cortactin phosphorylation and host cell membrane ruffling.^49^ The process of cellular invasion by *C. jejuni* has been coined the ‘binding and effector mechanism’.^50^ During the process of cellular invasion, *C. jejuni* stimulates the release of IL-8 (primarily from the basolateral surface) of cells.^51-53^ The increased concentration of IL-8 results in neutrophil influx, and ultimately leads to tissue damage (Figure 10). Although it is not clear where the neutrophils become activated, it is evident that there are activated neutrophils in the intestinal lumen based on the increase of CD11b (ITGAM) protein.^54^

A goal of this study was to determine whether the initiation of intestinal inflammation was dependent on the ability of *C. jejuni* to invade the IECs. In this regard, we found that the amount of both IL-8 and TNF-α cytokines in the intestinal lumen increased sooner after inoculation with the virulent *C. jejuni* wild-type strain compared to the *∆ciaD* mutant. This result is consistent with the finding that a *ciaD* deletion mutant caused less IL-8 production from THP-1 and T84 cells. Furthermore, CiaD itself may be participating in the IL-8 induction, as prior studies have shown that ectopic expression of CiaD-EGFP in human epithelial cells causes IL-8 production.^17^ We also utilized proteomic approaches to determine the host response to *C. jejuni* infection and found that neutrophil markers dominated the changes in the intestinal lumen. Macrophage-specific markers CSF1R, CD68, and CD163^55^ did not increase in the intestine due to *C. jejuni* inoculation. Using an *in vitro* model, intestinal epithelial cells were tested for their ability to indirectly signal to immune cells to prompt cytokine production. Relevant to the findings with the animal model, the ability of T84 epithelial cells to induce IL-8 secretion from THP-1 macrophage-like cells was dependent on the presence of CiaD and the ability of *C. jejuni* to invade cells. Using this newly developed pig model of *C. jejuni* disease, we have gained significant insight into the intestinal environment during the early process of infection and the process of initiating intestinal inflammation during *C. jejuni* infection.

Given that human disease symptoms develop 48–72 hours post-ingestion, we targeted earlier time points to determine the specific events that begin the inflammatory process. We determined that inflammatory cytokine induction (IL-8 and TNF-α) was most pronounced during inoculation with the invasive *C. jejuni* wild-type strain. These cytokines increased in concentration much earlier than the onset of disease symptoms, as soon as 12 hours after infection. Because epithelial cells primarily secrete IL-8 and TNF-α at the basolateral surface, the luminal IL-8 and TNF-α is either the result of a disruption of the intestinal barrier or produced from immune cells recruited to the lumen.^51,56^ In humans, IL-8 and TNF-α are produced from *C. jejuni*-specific CD4 + T-cells and are thought to further potentiate infection due to the promotion of inflammation.^57^ At 30 hours post-infection, there were mild histopathological lesions and an increase in IL-17A, which is a cytokine associated with a ‘leaky gut’ and appears to be associated with the development of inflammatory bowel syndrome and intestinal fibrosis.^58^ Specifically, IL-17A is produced by differentiated Th17 cells, which further points to the beginning of an adaptive immune response being mounted against the *C. jejuni*. Compared to the intestinal pathology, which is quite mild at 30 hours after infection, the intestinal cytokines were significantly altered as early as 12 hours after infection. This difference in timing suggests that *C. jejuni* causes intestinal inflammation very early during colonization, and further indicates that the disease outcomes of invasive and attenuated *C. jejuni* diverge well before the necrosis caused by *C. jejuni* intracellular replication.

Proteomic analysis of the intestinal contents revealed key neutrophil markers, and the specific host antimicrobial factors that are deployed by the host to combat *C. jejuni* infection. We used Gene ontology (GO) term enrichment analysis to determine if there were relevant protein functions enriched in the proteomic data set. The top categories of proteins that increased in abundance were ‘positive regulation of establishment of protein localization to telomere’ and ‘azurophil granule.’ Despite the category names, most of these proteins are involved in neutrophil degranulation. Furthermore, analysis of the data set with the Reactome pathway database identified ‘Neutrophil degranulation’ as the most highly enriched pathway. While neutrophils are a key mediator of the innate immune response, they are also a cause of intestinal damage during inflammatory responses.^59^ This inflammation-driven intestinal damage is hypothesized to potentiate further intestinal infection by disrupting the barrier between the intestinal epithelial cells.^60^ The proteome data ultimately points to neutrophil infiltration into the intestine. This trend has been observed in human infections where elevated levels of the neutrophil proteins S100A8, S100A9, and S100A12 are detectible in the stool.^61^ The S100A8 and S100A9 proteins are antimicrobial factors and potent neutrophil chemoattractants;^30,62^ these proteins also promote neutrophil activation, and S100A9 potentiates IL-8 production from neutrophils in response to other stimuli.^63^ In addition to these three proteins, our study has uncovered additional host factors that appear in the intestine during *C. jejuni* infection, including antimicrobial neutrophil related proteins.

The immune responses to *C. jejuni* appear to coincide with the ability of *C. jejuni* to invade epithelial cells. When the pig intestinal loops were infected with a *C. jejuni* isolate lacking CiaD, a protein that is important for cellular invasion and IL-8 induction,^17^ the amount of TNF-α in the lumen of the intestinal loops was lower compared to loops infected with the *C. jejuni* wild-type strain. To investigate the mechanistic basis for the observation, we infected human colonic epithelial cells (T84) and macrophage-like cells (THP-1) with the *C. jejuni* wild-type strain and the ∆*ciaD* mutant. Direct infection of both cell types (T84 and THP-1) with the *C. jejuni* wild-type strain resulted in high levels of IL-8 induction and lower IL-8 induction by the ∆*ciaD* mutant. Furthermore, cell-free conditioned medium from infected T84 cells was able to induce an IL-8 response in THP-1 cells that was nearly identical to direct infection of the THP-1 cells. This suggests that both direct and indirect actions by *C. jejuni* are able to modulate immune cell responses. This finding supports a model whereby the intestinal epithelial cells act as the front-line defense against *C. jejuni* and have the ability to initiate neutrophil influx. We propose that the initial influx of neutrophils potentiates further immune cell recruitment and intestinal inflammation. In other words, the early events during *C. jejuni* colonization determine the course of disease symptoms. Because the *C. jejuni* wild-type strain is capable of invading epithelial cells, it may use this strategy to avoid being killed by neutrophils recruited to the intestine by inflammatory cytokines. We propose that the *C. jejuni* ∆*ciaD* mutant, which is attenuated in invasion, lacks the ability to evade intestinal immune cells and is killed by these cells. This proposal is further supported by the finding that the decrease in luminal *C. jejuni* from 12 to 30 hours is greater in the ∆*ciaD* mutant than the wild-type strain.

Analysis of the *C. jejuni* within the intestine indicated unique *in vivo* responses to the pig ligated loop. More specifically, the early *C. jejuni* transcriptome indicated that *C. jejuni* was not responding to bile, but rather host cells and other intestinal components. It is possible that some of the *in vivo* responses, which included reduced expression of iron acquisition genes (*chuA, chuB, chuD, ceuB, cfbpA, cfbpB, cfrA*, and *cj1658*) and oxidative stress response genes (*katA*), were due to the intestinal mucus. It has been observed that incubating *C. jejuni* with mammalian mucus results in similar changes in gene expression when compared to avian mucus.^64^ In contrast, it has been found that in the chicken cecum, there is increased expression of *chuA, chuB, chuD*, and *katA* compared to growth in MH broth.^65^ This further indicates that *C. jejuni* initiates different transcriptional responses during mammalian disease compared to colonizing a commensal avian host.

In the present study, we have defined an important animal model to study the host and bacterial factors that initiate intestinal inflammation during *C. jejuni* infection. The key advantage of this model is the ability to study immune responses that are likely to mimic the human infection process. Using this system, we have found that *C. jejuni* induces an immune response as early as 12 hours after infection that is characterized by neutrophil influx, highlighting a key time during the infection process. Furthermore, the early immune responses in the intestine are dependent on a virulent *C. jejuni*. This data supports a model where virulent *C. jejuni* cause rapid cytokine production from resident intestinal cells (Figure 10), while an attenuated *C. jejuni* ∆*ciaD* mutant results in a delayed cytokine response. Using the immune signaling platform established by the proteomic evaluation of the intestinal loops, it will be possible to specifically target *C. jejuni* virulence determinants to determine the mechanisms of disease progression in the intestine.

## Materials and methods

### Bacterial strains

The *C. jejuni* F38011 wild-type strain was grown on Mueller-Hinton agar plates supplemented with 5% citrated bovine blood (MHB) or in MH broth in a microaerobic atmosphere (5% O_2_, 10% CO_2_, 10% H_2_, 75% N_2_) at 37°C. The *C. jejuni* ∆*ciaD* mutant and complemented isolates were generated as described previously.^17^ When needed, MHB agar plates were supplemented with 128 µg/mL of tetracycline or 8 µg/mL of chloramphenicol. The isolates were routinely passaged every 24 to 48 hours on MHB agar. Colony-forming unit (CFU) determination from fecal and intestinal samples (described below) was done by serially diluting the samples and plating on Campy-Cefex agar,^66^ a selective medium, and incubating for 48 to 72 hours.

### Pig acquisition and transport

This study was performed in accordance with the National Research Council’s *Guide for the Care and Use of Laboratory Animals* (8th edition) and approved by the Washington State University Institutional Animal Care and Use Committee under protocol ASAF 6236. Pigs (*Sus scrofa domesticus* or Minnesota mini pigs) of both sexes were obtained from the National Swine Research and Resource Center located at the University of Missouri-Columbia. All twelve pigs, three per experimental time point, were culture-negative for *C. jejuni* and *Campylobacter coli* prior to shipment, as judged by streaking fecal samples onto Campy-Cefex agar plates and incubating the plates at 37°C in a microaerobic atmosphere for 72 hours. At the time of arrival, the pigs were 20–23 weeks of age and weighed between 47 and 82 lbs. Pigs were given feed [Mazuri Mini Pig Active Adult (Land O’Lakes, Inc., Arden Hills, Minnesota)] and water *ad libitum*. The pigs were given oral ampicillin (20 mg/kg) 5 and 7 days prior to surgery to enhance *Campylobacter* colonization.^67^ At the time of the experiments, the pigs were between 27 and 39 weeks of age and between 70 and 90 lbs.

We chose to utilize Minnesota minipigs from the University of Missouri National Swine Resource and Research Center (NSRRC) after unsuccessful attempts to use locally sourced Yorkshire cross pigs. Regardless of the time of year (May to November), we were unable to obtain Yorkshire pigs locally that were free from *C. coli*. Although the amount of culturable *C. coli* in the stool was less than 1 × 10^4^ CFU/g and was reduced to undetectable levels by treatment with ampicillin, there were still detectable levels of *C. coli* in the distal region of the intestine by culture. In studies up to 24 hours in duration, we did not observe any *C. jejuni* induced disease symptoms in the locally sourced pigs that were *C. coli* positive. To avoid the confounding factor of resident *Campylobacter*, we obtained pigs that were free of resident *C. coli* from the NSRRC.

### Anesthesia

All animals were fasted from solid food for 12 hours before the surgery. For sedation, the pigs received an intramuscular injection of 0.05 mg/kg of dexmedetomidine (Dexdomitor® 0.5 mg/mL, Orion Corporation, Finland) and 10 mg/kg of ketamine (Ketaset® 100 mg/ml, Zoetis Inc., Spain). The animals were transported to the surgical area when non-responsive to handling. If more sedation was needed, an additional 5 mg/kg ketamine was given intramuscularly. The pigs were placed on the surgical table at sternal recumbency. A face mask was applied to the animal’s snout for additional muscle relaxation with 5% isoflurane (isoflurane USP, Akorn Inc, USA) in 100% oxygen. Promptly, the animals were orotracheally intubated with a cuffed endotracheal tube. Once the endotracheal tube was secured and pressure checked, it was connected to a mechanical ventilator (Tafonius junior, Hallowell EMC, USA) to maintain normocapnia. Anesthesia was maintained with isoflurane (1–2%) in 50% oxygen and 50% medical air delivered via a circle rebreathing system. During the entire duration of the anesthesia, the animals were monitored continuously using multiparametric monitoring equipment (DPM™6, Mindray DS, USA). The cardiovascular and pulmonary variables monitored were: heart rate via electrocardiography, respiratory rate, blood oxygen saturation, pulse rate, ETCO_2_, noninvasive arterial blood pressure (i.e., systolic, diastolic, and mean arterial blood pressures), and body temperature. A heating pad was used to maintain the pig’s body temperature above 98 °F (HotDog™ Veterinary Warming Controller, Augustine Biomedical and Design, USA). To maintain normotension, vasopressors were given at a constant rate of infusion when mean arterial blood pressure was less than 60 mmHg. The vasopressors available were dopamine 5–15 mcg/kg/min (dopamine HCl USP, 40 mg/mL, Hospira, USA), and norepinephrine 0.1–1 mcg/kg/min (norepinephrine bitartrate injection USP, 1 mg/mL, Claris, USA). Fluid therapy was provided with lactated Ringer’s solution (USP, Hospira, USA) at 10 mL/kg/hour for the entire duration of anesthesia. Blood glucose was monitored every 6 hours and maintained at levels above least 100 mg/dL by intravenous infusion of 2.5% dextrose (dextrose USP 0.5 g/mL, Hospira, USA). For analgesia, a lidocaine constant rate of infusion of 25–50 mcg/kg/minute was started after the administration of a 1 mg/kg lidocaine loading bolus intravenously (Lidocaine HCl injection USP, 2%, Hospira, USA). After the indicated duration (from 3 to 30 hours), the animal’s small intestine was surgically removed. Euthanasia was performed with an intravenous injection of pentobarbital sodium and phenytoin sodium at 1 mL for every 4.5 kg of body weight (Euthasol, Virbac AH, Inc., USA).

### Surgery

The pigs were positioned in dorsal recumbency, and following a standard surgical scrub, a 7 cm ventral midline celiotomy was performed to expose the intestine. Prior to the loop construction, the lumen of the ileum and distal jejunum was gently washed with a phosphate-buffered saline (PBS) injection to remove the intestinal contents. Ligated intestinal loops were constructed starting 10 cm proximal of the ileocecal junction using monofilament suture. Ligation was done by a circumferential ligature through the mesentery without damaging grossly visible mesenteric vascular arcades, thus maintaining full blood supply for both the *C. jejuni-*inoculated loops and control (inter-loop) segments. *C. jejuni* was grown to mid-log phase, and the cells pelleted by centrifugation. The cells were resuspended in PBS to an OD_540_ of 0.35. Loops were inoculated with approximately 1 × 10^10^ bacterial cells suspended in 10 mL of PBS using a 21-gauge needle. The inter-loop segments were mock-inoculated with sterile PBS as a negative control. Care was taken to avoid over-distension of loops. A 16 G, 5.25-inch catheter was surgically placed in the urinary bladder to drain the bladder during the incubation period and then attached to a fluid extension to monitor the urinary output of the animal. The abdominal incision was closed, and the pigs were maintained under anesthesia for the remainder of the experiment. After incubation, the abdominal incision was opened, and the ligated section of the intestine was removed. The luminal contents and intestinal tissues were recovered and processed for further analysis. The animals were euthanized, as outlined above.

### Sample collection

Luminal contents, mucosal scrapings, and intestinal tissues were collected and processed for analysis. All loops were subjected to gross and histopathologic evaluation, plating of serial dilutions (to measure bacterial numbers), measurement of cytokine secretion, proteomic analysis, and analysis of the microbiota associated with the luminal contents and tissues. Loops were separated by cutting between the double ligation. Luminal contents were collected by injecting each loop with 5 mL of PBS and collecting the resulting fluid. The fluids were centrifuged at 14,000 x *g* for one minute to collect the supernatants and pellets. Tissue scrapings were collected from a 1 inch^2^ tissue section using a tissue-culture scraper. Mucosa was suspended in 5 mL of PBS and collected by centrifugation. Processed luminal contents and tissue scrapings were flash-frozen in liquid nitrogen immediately after collection. Tissue samples for histology were immediately transferred to 10% neutral buffered formalin for fixation before histochemical processing.

### IPEC-J2 cell culture and RNA-Seq sample collection

The IPEC-J2 cells were kindly provided by Dr. Anthony Blikslager at North Carolina State University.^68^ The IPEC-J2 cells were routinely cultured in a 1:1 mixture of Dulbecco’s modified Eagle medium and Ham’s F12 Nutrient Mixture (DMEM/F12) supplemented with 10% fetal bovine serum (FBS; GE Healthcare Life Sciences, HyClone Characterized Fetal Bovine Serum US Origin cat. # SH30071), 1% insulin-transferrin-selenium supplement (Thermo Fisher Scientific, Waltham MA), and 2.5 µg/mL epidermal growth factor (Thermo Fisher Scientific). The samples for RNA-Seq analysis were generated as follows. *C. jejuni* were first inoculated at an OD_540_ of 0.05 in MH broth and incubated with shaking for 18 hours. A liquid culture of *C. jejuni* was then adjusted to an OD_540_ of 0.3 in DMEM/F12 supplemented with 1% FBS and incubated with the IPEC-J2 cells for 2.5 or 4 hours at 37°C with 5% CO_2_. The sample used to infect the cells was collected as the ‘input’ sample. The supernatant was collected, and the bacteria were recovered by centrifugation and collected in 1/10 volume of ice-cold stop solution (5% phenol, 95% ethanol), flash-frozen in liquid nitrogen, and stored at −80°C until RNA extraction. For IPEC-J2 samples, RNA was processed as described previously ^22^. RNA from pig loops was extracted with the SV Total RNA Isolation System (Promega) with 3 mg/mL lysozyme, treated with Turbo DNAse AM1907 (Thermo Fisher), and rRNA was depleted using 1/8th reaction volumes of Ribo-Zero Gold rRNA Removal Kit-Epidemiology (Illumina, San Diego, CA) following manufacturer’s instructions with ethanol purification.

### RNA-Sequencing

Illumina MiSeq libraries were prepared using the KAPA stranded RNAseq kit (Kapa Biosystems, Wilmington, MA), following the manufacturer’s instructions except for the following changes: 1–187 ng RNA was sheared for 6 min at 85°C. Standard desalted TruSeq HT primers (Integrated DNA Technologies, Coralville, IA) were used at 25–50 nM final concentration based on starting RNA amount. The PCR step was performed for six to thirteen cycles. Libraries were quantified using the KAPA Library Quantification Kit (Kapa), except with 10 µL volume and a 90 second annealing/extension PCR. Libraries were pooled and normalized to 4 nM. The pooled libraries were re***‐***quantified by ddPCR on a QX200 system (Bio***‐***Rad, Hercules, CA) using the Illumina TruSeq ddPCR Library Quantification Kit and following manufacturer’s protocols. The libraries were sequenced in two 2 × 76 bp paired-end v3 runs on a MiSeq instrument at 13.5 pM, following manufacturer’s protocols. Fastq files were generated for each sample by the MiSeq Instrument Software. Reads were mapped to the reference genome of *C. jejuni* subsp. *jejuni* F38011 (CP006851) using Bowtie2 (version 2.2.5) and reads were assigned to genes using featureCounts (version 1.5.0). Differential expression analysis was performed using DESeq2 (version 1.10.1).

To construct the hierarchical clustering of expression data from the pig, deoxycholate (DOC), and IPEC-J2 cells, the data were analyzed where each individual sample was compared to its respective control (input) sample grown in MH broth. The data were filtered such that genes were only kept if they had a Benjamini-Hochberg adjusted *p*-value (*q*-value) of ≤ 0.05, and had a log_2_(fold change) of greater than 1.5 (or less than −1.5) in at least four of the six compared samples (two time points per condition). These filters were established to select the genes that are significantly and meaningfully changing in our samples, and to find genes that were changing at both time points in each of the samples.

### Determination of cytokine levels

TNF-α, IL-8, and IL-17A concentrations were assessed in *C. jejuni*-infected and non-infected samples collected from pig intestinal ligated loops. Cytokine levels were analyzed by using commercially available enzyme-linked immunosorbent assay (ELISA) kits (R & D systems cat #s PTA00 and P8000, and Thermo Fisher Scientific cat # ESIL17A). All ELISAs were performed according to the manufacturer’s protocol.

### Microbiome analysis

Genomic DNA was isolated from mucosal and luminal samples using the MagAttract PowerMicrobiome 96-well DNA/RNA kit (Qiagen, Germantown, MD) following the manufacturer’s instructions. The V4 region of the bacterial 16S rRNA gene was PCR amplified and sequenced on the MiSeq platform (Illumina) as previously described.^69^

The 16S rRNA gene sequences were processed in R version 3.5.1 using the Dada2 package version 1.8.0.^70^ Forward reads were truncated at 220 bp and reverse reads at 210 bp. A maximum expected error of 2 was assigned for both paired reads with no ambiguous bases allowed. Reads were merged, chimeric sequences removed, and taxonomy assigned at 100% similarity using the naive Bayesian RDP classifier^71^ and the SILVA SSU database release 132^72^ with a 50% bootstrap confidence threshold. Although control loops were confirmed to be *Campylobacter* negative after selective culture (see above), some *Campylobacter* 16S rRNA gene reads were detected in some control samples. Because of cross-contamination between adjacent samples in 96-well DNA extraction formats has been previously observed,^73^ as well as sequencing barcode bleed-through in multiplexed sequencing runs,^74^ only control samples with 0.5% or fewer *Campylobacter* reads were retained for analysis. Ordination plots were made with PRIMER software (v7).^75^

### Proteomics sample preparation

Both intestinal pellets and supernatants were analyzed by LC-MS/MS for protein content. Proteins in the supernatant samples were purified following the Metabolite, Protein and Lipid Extraction (MPLEx) method as described previously.^76^ In brief, samples were mixed with cold (−20°C) chloroform and methanol at a volume ratio of 10:5:3. The mixture was chilled on ice for 5 minutes, vortexed for 1 minute, and centrifuged for 10 minutes at 4°C at 16,260 × g. The interphase that contained the proteins was collected and washed twice with cold (−20°C) methanol. Recovered protein pellets were then denatured in 100 μL of 8 M urea in 50 mM NH_4_HCO_3_ (pH 8.0). Dithiothreitol (DTT, Thermo Fisher Scientific) was added to a final concentration of 5 mM prior to incubation at 60°C for 30 minutes with shaking at 850 rpm. After DTT reduction, iodoacetamide (IAA, Thermo Fisher Scientific) was added to a final concentration of 40 mM to alkylate free thiol groups, followed by incubation at 37°C for 1 hour with shaking at 850 rpm in the dark. Samples were then diluted ten-fold with 100 mM NH_4_HCO_3_ (pH 8.0) and adjusted to a CaCl_2_ concentration of 1 mM. Trypsin was added at a 1:50 enzyme-to-protein ratio to digest proteins at 37°C for 3 hours. The digestion was stopped by adding trifluoroacetic acid to a concentration of 0.1%, followed by centrifugation at 10,000 g for 2 minutes to remove precipitates. Peptides were cleaned using DSC-18 solid phase extraction tubes (Supelco Inc., Bellefonte, PA) and dried in a SpeedVac vacuum concentrator. The final peptide concentration was quantified by a Pierce^TM^ BCA protein Assay Kit (Thermo Fisher Scientific) and adjusted to 0.1 μg/μL with Milli-Q water for proteomic analysis.

Pellet samples were resuspended in 200 μL of 50 mM NH_4_HCO_3_ (pH 8.0) and subjected to bead beating with 0.1 mm Zirconia/Silica beads (BioSpec Products, Bartlesville, OK) using five cycles consisting of a 1 minute vortex with a 30 second break. After bead beating, samples were moved to new tubes and mixed with powdered urea to a final concentration of 8 M. The denatured samples were mixed with DTT to a final concentration of 10 mM and incubated at 37°C for 1 hour with shaking at 750 rpm. The samples were then mixed with IAA to a final concentration of 40 mM and incubated at 37°C for 1 hour in the dark. Samples were diluted ten-fold with 50 mM NH_4_HCO_3_ (pH 8.0) containing 1.1 mM CaCl_2_. Trypsin was added at a 1:50 enzyme-to-protein ratio, and the mixture was incubated at 37°C for 3 hours. Digested peptides were cleaned using DSC-18 solid phase extraction tubes, dried in a SpeedVac vacuum concentrator, and finally adjusted to 0.1 μg/μL for proteomic analysis.

### Proteomics analysis

The digested proteins were analyzed with liquid chromatography-tandem mass spectrometry (LC-MS/MS). The liquid chromatography (LC) consisted of a Waters nanoAcquity UPLC system equipped with a custom analytical column packed with 3 µm Jupiter C_18_ media (Phenomenex, Torrance, CA) into 70 cm × 75 µm (ID) fused silica (Polymicro Technologies, Phoenix, AZ). 5 µL of each digested sample was loaded onto the LC system, and peptides were separated at 300 nL/minute at room temperature. Mobile phases comprised mobile phase A (0.1% formic acid in water) and mobile phase B (0.1% formic acid in acetonitrile). The supernatant peptides were separated with the following gradient: 1–8% B for 2 minutes, 8–12% B for 18 minutes, 12–30% B for 55 minutes, 30–45% B for 22 minutes, 45–95% B for 3 minutes, maintained at 95% for 10 minutes, and equilibrated at 1% B. The pellet peptides were analyzed with the following gradient: 1–8% B for 4 minutes, 8–12% B for 32 minutes, 12–30% B for 96 minutes, 30–45% B for 40 minutes, 45–95% B for 5 minutes, maintained at 95% for 10 minutes, and equilibrated at 1% B.

The mass spectrometry (MS) analysis was conducted in a Q Exactive Plus mass spectrometer (Thermo Fisher Scientific) outfitted with a custom nano-electrospray ionization (ESI) interface.^77^ Spray voltage and capillary temperature were controlled at 2 kV and 325°C, respectively. Full MS scans were acquired from 300–2000 m/z at a resolution of 35,000. Top 12 data-dependent tandem MS scans were performed at a resolution of 17,500, isolation window of 2.0 m/z, normalized collision energy of 30, and dynamic exclusion of 30 seconds.

### Proteomics data analysis

The supernatant and pellet RAW datafiles were searched separately with MaxQuant (v1.6.0.16)^78^ against a database comprising the Uniprot *Sus scrofa* proteome and *C. jejuni* F38011 proteome. Carbamidomethylation was set as a fixed modification, and methionine oxidation and N-term acetyl were set as variable modifications. A matching window within 1.5 minutes was applied to the samples. False discovery rate (FDR) at the peptide level and protein level was set less than 0.01. Trypsin was set as the digestion enzyme. Default settings were used for the rest of the MaxQuant search parameters. Proteins were quantified using label-free quantification.

The MaxQuant output containing proteins and corresponding label-free quantification was processed with R package RomicsProcessor v0.1.0, which is available on both Github (https://github.com/PNNL-Comp-Mass-Spec/RomicsProcessor) and Zenodo (https://zenodo.org/record/3386527). Briefly, the protein features were filtered to retain proteins with more than 80% of detection among biological replicates under infected conditions or control conditions. The remaining missing values were imputed with the underlying assumption that values were distributed typical of low abundances levels.^79^ In the statistical analysis, Student’s paired *t*-test was applied to determine if the mean abundance of a protein was different in the infected loops and in the control loops. The infected loops were paired with their adjacent control loops. The null hypothesis was that the mean protein abundances were the same was rejected if the *p*-value was less than 0.05. The proteins with significantly different expression levels (*p* < .05) had enrichment analyses performed for Gene Ontology (GO) terms, KEGG pathways, and Reactome pathways with an online tool g:Profiler.^80^ The log_2_ fold change of protein abundance due to infection was calculated by normalizing the mean protein abundance in the infected loops to the mean protein abundance in the control loops.

### T84 and THP-1 cell culture

T84 cells (ATCC CCL-248) were routinely cultured in Eagles MEM (Gibco, Waltham, MA) supplemented with 10% FBS (Cat# 97068–091, VWR, Radnor, PA) at 37°C in a 5% CO_2_ incubator. THP-1 cells were maintained as described previously.^81^ Three days prior to the experiments, the THP-1 cells were differentiated using 100 nM phorbol 12-myristate 13-acetate (PMA; Sigma Aldrich) treatment for 24 hours.

### Determining adherent and internalized C. jejuni

The number of adherent and internalized *C. jejuni* was determined as described previously.^82^ Briefly, the number of adherent bacteria was determined by infecting T84 cells in a 24-well dish with *C. jejuni* at an OD_540_ of 0.03 in MEM supplemented with 1% FBS and incubating for three hours at 37°C in a 5% CO_2_ incubator. The cells were rinsed with PBS three times, lysed with 0.1% Triton X-100, and lysates were serially diluted and plated on MHB agar. To determine the number of internalized bacteria, T84 cells were infected with *C. jejuni* for three hours, rinsed three times with PBS, and then treated with 250 µg/mL gentamicin for three hours. The cells were then rinsed, lysed with 0.1% Triton X-100, and lysates were serially diluted and plated to determine the number of bacteria that survived the gentamicin treatment.

### Detection of human IL-8 cytokines from cultured cells

Cultured cells, either T84 or THP-1, were treated with *C. jejuni* at an OD_540_ of 0.03 or conditioned medium for 24 hours. The supernatants were collected and centrifuged at 13,000 x *g* for 5 minutes to remove any cells or cell debris. The amount of IL-8 in the supernatant was determined by a commercial ELISA kit following the manufacturer’s instructions (Cat# 555244, BD Biosciences, San Jose, CA).

### Data availability

RNA-Seq data is available at the NCBI Gene Expression Omnibus database with the identifier GSE147629. Proteomics data is available at MassIVE (Mass spectrometry Interactive Virtual Environment) with accession number MSV000079501. The 16S rRNA gene sequence data is available under the NCBI BioProject PRJNA615040.

## Supplementary Material

Supplemental Material
