# Supplementary material for: A porcine ligated loop model reveals new insight into the host immune response against Campylobacter jejuni
Source: Gut Microbes. 2020 Sep 4;12(1):1814121. doi: 10.1080/19490976.2020.1814121 (PMC7524355; doi:10.1080/19490976.2020.1814121)

Supplementary Figure 1. There was no significant correlation between cytokine levels and loop position or experimental animal. Cytokine levels for individual animals were plotted by loop position, where position 1 is closest to the ileocecal junction (first loop inoculated). Incubation time is indicated by color (blue = 3 hours, red = 6 hours, green = 12 hours), and the horizontal bar represents the mean of the samples at that given time. Panels: **A:** The amount of IL-8 measured in control loops. **B:** The amount of IL-8 measured in *C. jejuni*-inoculated loops, where loops 1 and 3 were inoculated with the *C. jejuni* wild-type strain and loops 2 and 4 were inoculated with the *C. jejuni* ∆*ciaD* mutant. **C:** The amount of TNF-α measured in control loops. **D:** The amount of TNF-α measured in *C. jejuni*-inoculated loops, where loops 1 and 3 were inoculated with *C. jejuni* wild-type strain and loops 2 and 4 were inoculated with the *C. jejuni* ∆*ciaD* mutant.

Supplementary Figure 2. An expanded model based off the proteomic analysis was developed. This model includes proteins from the intestinal pellets and supernatants that significantly increased in abundance (*p*-value of ≤ 0.05 and a log_2_ fold change > 0) in intestinal loops inoculated with the *C. jejuni* wild-type strain compared to non-infected loops. In addition, this model includes proteins that were detected in the intestinal lumen but did not significantly change. Proteins that increased in abundance were involved in neutrophil function: Integrins (α_M_β_2_), CD177, Arp2/3, RAB7A, and NOS2. Additionally, neutrophil-related secreted proteins were also increased: S100A8, S100A9, S100A12, LCN2, MMP9, PRTN3, and neutrophil elastase (ELANE). Proteins that are involved in actin reorganization were detected in the intestinal lumen including: CDC42, RAC, RHO, MAPK, and IQGAP.


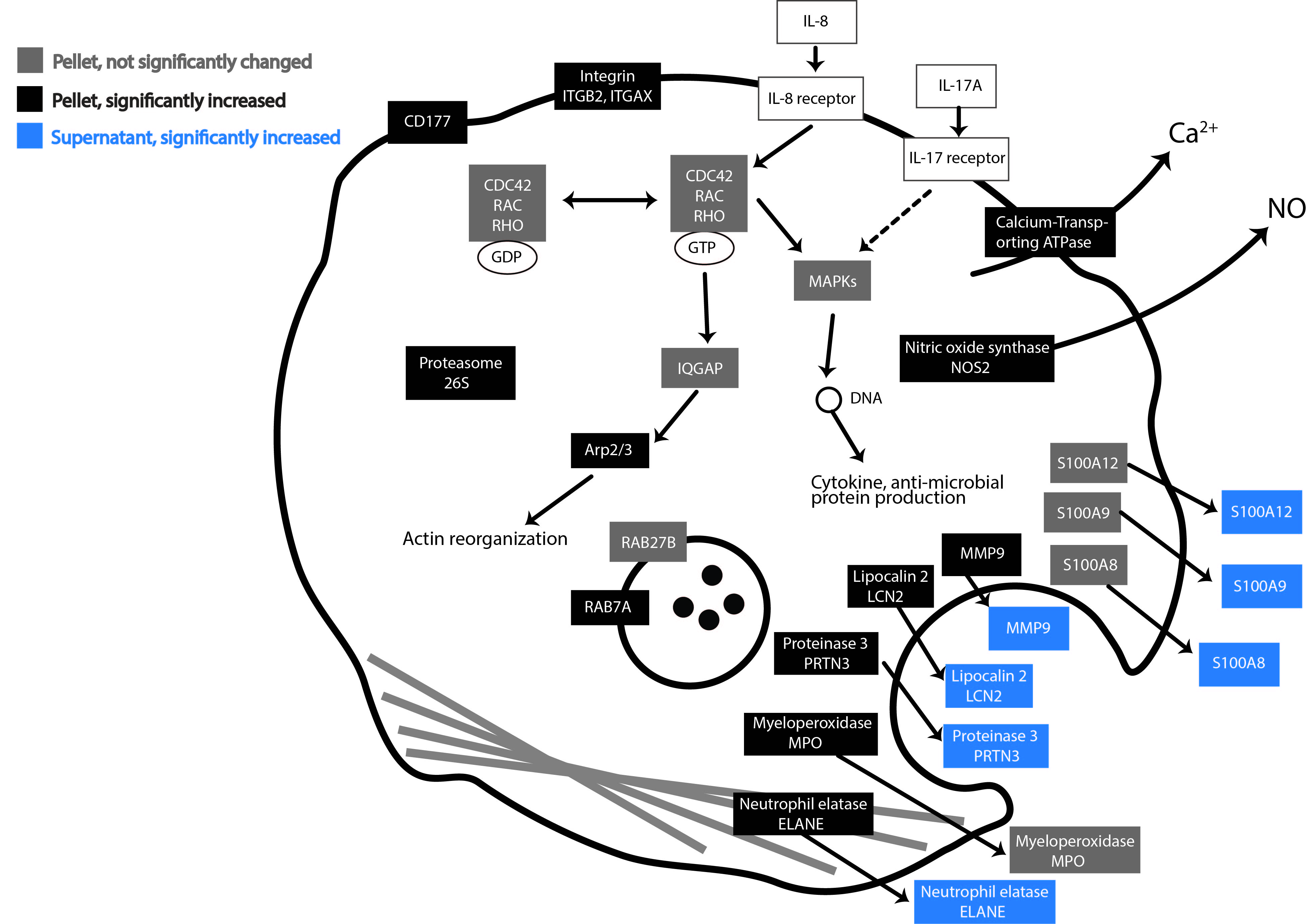

Supplement: Supplemental Material [file KGMI_A_1814121_SM1734.zip › Supplementary information/Supplementary Figures.docx]
